# Draft Genome Sequence of *Lactobacillus* sp. Strain TCF032-E4, Isolated from Fermented Radish

**DOI:** 10.1128/genomeA.00821-15

**Published:** 2015-07-30

**Authors:** Yuejian Mao, Meng Chen, Philippe Horvath

**Affiliations:** a DuPont Nutrition & Health, Shanghai, China; b DuPont Nutrition & Health, Dangé-Saint-Romain, France

## Abstract

Here, we report the draft genome sequence of *Lactobacillus* sp. strain TCF032-E4 (= CCTCC AB2015090 = DSM 100358), isolated from a Chinese fermented radish. The total length of the 57 contigs is about 2.9 Mb, with a G+C content of 43.5 mol% and 2,797 predicted coding sequences (CDSs).

## GENOME ANNOUNCEMENT

*Lactobacillus* sp. strain TCF032-E4 was isolated in 2013 from a homemade fermented white radish (brine pH 3.7) originating from Guilin, Guangxi Province, China. The 16S rRNA gene sequence of this strain shared high similarity with that of *Lactobacillus plantarum* subsp. *plantarum* ATCC 14917^T^ (99.2%), *Lactobacillus pentosus* NCDO 363^T^ (99.2%), and *Lactobacillus paraplantarum* CNRZ 1885^T^ (99.1%). However, an abnormal amplification pattern (no product) was obtained with the *recA* multiplex PCR, a method described by Torriani et al. (1) for the differentiation of *L. plantarum, L. paraplantarum*, and *L. pentosus* species, which share highly similar 16S rRNA genes. TCF032-E4 also showed a different growth temperature range (no growth at temperatures of >32°C) and sugar fermentation pattern (e.g., no sucrose utilization). These distinguishing phenotypic characters motivated a deeper analysis of TCF032-E4 through whole-genome sequencing.

The genome of *Lactobacillus* sp. TCF032-E4 was sequenced by Majorbio Biopharm Technology Co., Ltd. (Shanghai, China) using an Illumina HiSeq 2500 platform. A paired-end library with a DNA insert length of 300 bp was generated, and 11,896,210 reads with a mean length of 101 nucleotides (nt) were obtained, representing ~413-fold genome coverage. Velvet 1.2.10 (2) was used to assemble the reads using a *k*-mer value of 31 (-ins_length 300, −cov_cutoff 50, -exp_cov 410, -min_contig_lgth 500). The resulting draft genome sequence comprised 57 contigs >500 bp in length, with a maximum contig length of 279,565 bp. The total length of all contigs is 2,904,660 bp, with a G+C content of 43.5 mol%. The contigs were ordered with Mauve 2.3.1 (3) using the complete genome of *L. plantarum* WCFS1 (4) (accession no. AL935263) as the reference, which shared 99% 16S rRNA gene similarity with TCF032-E4. The reordered draft genome sequence of TCF032-E4 was annotated with RAST (5), a fully automated online service for genome annotation. In total, 2,797 protein-coding sequences (CDS) were predicted, with an average length of 867 bp, representing a coding density of 83.5%. A total of 792 CDSs (28.3%) were identified to code for hypothetical proteins, while 1,225 CDS (44%) were classified into the RAST subsystems. Fifty-four tRNAs and one putative bacteriocin gene were predicted. Two intact prophages (45.3 kb and 34.4 kb) were detected by PHAST (6). No clustered regularly interspaced short palindromic repeat (CRISPR) array was detected using CRISPRFinder (7).

### Nucleotide sequence accession numbers.

The strain is publicly available from two collections under references CCTCC AB2015090 and DSM 100358. This whole-genome shotgun project has been deposited at DDBJ/EMBL/GenBank under the accession no. LFEE00000000. The version described in this paper is version LFEE01000000.
